# Time–energy budgets outperform dynamic body acceleration in predicting daily energy expenditure in kittiwakes, and estimate a very low cost of gliding flight relative to flapping flight

**DOI:** 10.1242/jeb.247176

**Published:** 2024-11-07

**Authors:** Fred Tremblay, Emily S. Choy, Shannon Whelan, Scott Hatch, Kyle H. Elliott

**Affiliations:** ^1^ Department of Natural Resources, McGill University, Montreal, QC, Canada, H9X 3V9; ^2^ Department of Biology, McMaster University, Hamilton, ON, Canada, L8S 4L8; ^3^ Institute for Seabird Research and Conservation, Middleton Island, AK 99516, USA

**Keywords:** Accelerometry, Energetics, Doubly labelled water, Activity budgets, *Rissa tridactyla*, Seabirds

## Abstract

Energy is a common currency for any living organism, yet estimating energy expenditure in wild animals is challenging. Accelerometers are commonly used to estimate energy expenditure, via a dynamic body acceleration (DBA) or time–energy budget approach. The DBA approach estimates energy expenditure directly from acceleration but may lead to erroneous estimates during inactivity when acceleration is zero but energy expenditure is not. The time–energy budget approach uses accelerometers and other data streams to assign a behaviour to each time step, and then calculates energy expenditure based on activity-specific metabolic rates assigned to each behaviour. Here, we used GPS-accelerometry in breeding black-legged kittiwakes (*Rissa tridactyla*, *n*=80) to calculate DBA and time–energy budgets derived from simple biologging metrics (speed, wingbeat frequency, GPS position). We then compared these two approaches with estimates of energy expenditure from doubly labelled water (DLW). Energy expenditure estimated from DLW correlated with DBA, but the best model to estimate energy expenditure was based on time–energy budgets. Energetic costs of flapping flight were higher than all other kittiwake behaviours (5.54×basal metabolic rate, BMR). Energetic costs of gliding flight (0.80×BMR) were the lowest of all behaviours, and equivalent to the cost of resting at the colony. DEE for our birds estimated from our calibration coefficients was similar to DEE for our birds estimated with the model coefficient published using different methods. We conclude that once calibrated with DLW, GPS-accelerometry provides a simple method for measuring energy expenditure in wild kittiwakes based on time–energy budgets.

## Teaser

**Summary:** Time–energy budgets outperform dynamic body acceleration in predicting daily energy expenditure in black-legged kittiwakes, and estimate a very low cost of gliding flight relative to flapping flight using simple and easily obtainable GPS-accelerometry metrics.

## INTRODUCTION

Energy is a limited resource; thus, understanding how energy is allocated to various life-history traits is critical to understanding the ecology of a species (Speakman, 1997; Butler et al., 1992, 2004). In particular, energy allocation is balanced between survival and reproduction, and individuals are constantly weighing the pros and cons of investing in their current or future reproductive success (Forbes et al., 2016; Wikelski and Cooke, 2006; Halsey et al., 2009). Multiple studies have investigated the effects of breeding and specific breeding stages on energy expenditure, showing that breeding stage has a significant impact on birds' energy expenditure (Golet and Irons, 1999; Dunn et al., 2020; Tremblay et al., 2022). Nonetheless, measuring energy expenditure itself remains a challenge for wild animals, especially for flying animals (Elliott et al., 2013a; Hicks et al., 2017).

Energy expenditure can be measured using methods such as doubly labelled water (DLW; Speakman, 1997), heart rate loggers (Butler et al., 2004) and accelerometry and accelerometer-derived time–energy budgets (Stothart et al., 2016; Wilson et al., 2006; Fort et al., 2011; Halsey et al., 2011). Although the DLW technique has been validated in many species, DLW can only measure expenditure over a relatively short period of time (hours to days) and provides a single value of energy expenditure (Butler et al., 2004; Green, 2011). Alternatively, heart rate loggers allow researchers to obtain estimates of energy expenditure over longer periods of time, yet the invasiveness of this method makes it less accessible to some researchers (Butler et al., 2004; Green, 2011). Accelerometers potentially allow for a less invasive approach to track complex behaviour and continuously monitor energy expenditure remotely, and their use has increased significantly in a wide range of species in the last decade as a result of technological advancements (Patterson et al., 2019; Fort et al., 2011; Elliott, 2016; Halsey et al., 2011). Once calibrated against DLW measurements of energy expenditure, accelerometry-derived metrics, such as dynamic body acceleration (DBA) or accelerometry-derived time–energy budgets, allow researchers to obtain estimates of energy expenditure on different time scales. Thus, this technique expands the period available to measure energy costs from a few days (DLW) to anywhere from a few seconds to years. Although DBA, an integrated metric of body movement in all three dimensions obtained from accelerometers (Halsey et al., 2009), has been used as a proxy for energy expenditure in a range of species, this method loses accuracy and precision when applied to species that spend a significant amount of time conducting low movement activities or where environmental DBA may be significant (i.e. resting on water or gliding; Fort et al., 2011; Jeanniard-du-Dot et al., 2017; Halsey and Bryce, 2021; but see Duriez et al., 2014). For such species, time–energy budgets can be better estimates of daily energy expenditure (Fort et al., 2011; Jeanniard-du-Dot et al., 2017; Wilson et al., 2020), with even simple active–inactive time budgets sometimes explaining most of the variation in energy expenditure (Studd et al., 2020). Time–energy budgets can provide valuable insights into an individual's energy expenditure, if activity-specific metabolic rates are known or can be estimated for the species of interest (Collins et al., 2016; Fort et al., 2011; Dunn et al., 2023).

Using accelerometry data to generate time–energy budgets can however be challenging in some instances as the frequency at which data points are recorded (often ranging from 20 to 100 Hz for birds; Shepard et al., 2008) creates computational and analytical challenges (Patterson et al., 2019; Williams et al., 2020). Despite this, the accelerometer metrics and choice of methods to extract behaviour have little impact on the accuracy of behavioural classifications of seabirds, including black-legged kittiwakes (*Rissa tridactyla*) (Patterson et al., 2019). In the context of time–energy budgets then, the key driver is to separate an animal's behaviours into broad categories that are the most likely to differ in their energetic costs (i.e. resting, swimming, flying, diving, etc.; Collins et al., 2016). Therefore, the use of broad behavioural categories that can be obtained using a few, easily calculated accelerometry metrics, such as wingbeat frequency, should be favoured to promote the method's accessibility, while still producing reliable time–energy budgets (Collins et al., 2016). Moreover, pairing biologging devices such as accelerometry with GPS can enable researchers to exploit the strengths of both devices (Williams et al., 2020), such as the computation speed of GPS data paired with the high resolution of more easily calculable accelerometry metrics (i.e. wingbeat frequency) to obtain reliable time–energy budgets (Patterson et al., 2019), which can be converted into energy budgets for discrete activities such as flapping and gliding flight.

Flapping flight is the most expensive form of sustained locomotion among vertebrates, with costs ranging up to 31×basal metabolic rate (BMR) (Videler, 2006; Elliott et al., 2013a). In contrast, gliding flight is less expensive than rest for some albatrosses (Bevan et al., 1995). Indeed, gliders have costs of flight that are roughly 10 times lower than those of flappers (Videler, 2006; Elliott et al., 2013a). Estimates on wild animals have been from studies classifying species as ‘flappers’ or ‘gliders’, yet most birds use a combination of flapping and gliding flight. Whereas some species rapidly alternate between flapping and gliding (i.e. flap-gliders such as sulids or shearwaters, or bounders such as finches), other species can have distinct flight modes, spending extensive time either gliding or flapping (i.e. gulls, raptors). The energetic costs of flapping and gliding are likely to be highly divergent, making it important to identify both the incidence and costs of these behaviours in the context of a time–energy budget. Yet to date, these costs have not been quantified using DLW in a free-ranging species that has such mixed flight. Because the metabolic states are distinct, it should be possible to estimate the cost of flapping and gliding using DLW, with a multiple regression that includes both gliding and flapping flight. Such estimates may describe the considerable unexplained variance in allometric relationships between body mass and flight costs that may arise because many ‘flapping flyers’ have periods of interspersed gliding flight (Videler, 2006; Guigueno et al., 2019).

Here, we derived activity-specific metabolic rates from DLW paired with simple accelerometry metrics and GPS data in breeding black-legged kittiwakes, a species that uses both flapping (primarily commuting) and gliding (primarily soaring near the colony) flight. Black-legged kittiwakes (hereafter kittiwakes) are a well-studied species that frequently engage in inactive behaviours such as gliding and resting (Collins et al., 2015, 2016). Kittiwakes, widespread sub-Arctic seabirds, are suitable candidates when it comes to studying energy expenditure using time–energy budgets as their behaviours can be summed up in a few coarse categories with divergent energetic costs (i.e. flapping, gliding, swimming and resting; Collins et al., 2015, 2016) and they are often used as a model species for gulls and seabirds because of their resilience to handling and wide-ranging abundance. Yet, published values of activity-specific metabolic rates are based on behavioural classifications at such a fine scale that such behaviours cannot be derived from biologging data (i.e. Jodice et al., 2003), or to a scale that is so coarse they pool energetically distinct behaviours into a single behaviour (i.e. ‘flight’; Collins et al., 2016; Tremblay et al., 2022). Pairing GPS and accelerometry together can distinguish behaviours that are energetically divergent but have been treated together in time–energy budget models, such as flapping and gliding flight (i.e. Collins et al., 2016; Tremblay et al., 2022).

We hypothesized that (1) time–activity budgets would better predict energy expenditure than DBA, as they are a better predictor of energy expenditure for species that frequently engage in inactive behaviours (i.e. gliding flight) and (2) time–energy budgets would yield more robust estimates of energy expenditure when gliding and flapping flight are separated into two distinct behavioural categories because of the high cost of flapping relative to gliding flight. To test our hypotheses, we estimated energy expenditure using DLW and deployed GPS-accelerometers on kittiwakes at a well-studied colony on Middleton Island, AK, USA, during the breeding season (including pre-laying, incubation and chick rearing). We developed simple accelerometry metrics and paired them with GPS data to classify behaviours into energetically divergent categories (flapping flight, gliding, resting on water, resting on land, attending the colony) and obtained precise time–energy budgets. We compared estimates of daily energy expenditure (DEE) from this approach with other estimates for this species and investigated the change in DEE between stages of the breeding season. As DEE and BMR vary by breeding stage in kittiwakes (Bech et al., 2002; Tremblay et al., 2022), it is important to account for stage-related differences to fully test both hypotheses, and we examined how DEE and time budgets change with stage. To determine whether our approach improves upon those existing in the literature, we compared our models with those using fine-scale (but poorly defined via radio telemetry) time budgets (Jodice et al., 2003) or coarse scale behaviours with energy costs estimated from modelling (Collins et al., 2016). Finally, we compared estimates of the energetic costs of flapping and gliding flight in kittiwakes with those from other species.

## MATERIALS AND METHODS

### Study design

We studied black-legged kittiwakes, *Rissa tridactyla* (Linnaeus 1758), breeding on the outside of an old radar tower retrofitted with one-way windows on Middleton Island, AK, USA, thus giving the researcher close access to monitor birds from the inside, with minimal disturbance. The one-way windows allowed us to visually identify individuals based on their unique combination of colour bands, and identify sex based on behaviour (i.e. copulation event). As we were able to identify sex in the field, we focused on males only to reduce individual variation in energy expenditure as it has been shown that females exhibit high individual variation in pre-laying, likely as a result of egg formation (Tremblay et al., 2022). As breeding stage influences energy expenditure (Tremblay et al., 2022), we included kittiwakes in pre-laying, incubation and chick-rearing stages. We captured birds using a leg hook, weighed them using a spring scale (Pesola, Baar, Switzerland; 500±2 g) and deployed GPS-accelerometers on two groups of kittiwakes: control birds which included pre-laying (*n*=10), incubating (*n*=10) and chick-rearing (*n*=18) kittiwakes, and DLW birds which included pre-laying (*n*=20) and incubating (*n*=35) kittiwakes. For both groups (controls and DLW birds), we fixed a GPS-accelerometer on the two central tail feathers (rectrices, including some coverts to protect the rectrices from damage) using super glue, Tesa tape and two zip ties (https://www.youtube.com/watch?v=bjlRK1pSxa8). We programmed the GPS-accelerometers to obtain GPS coordinates every 3 min and measure triaxial acceleration at 25 Hz. We released the birds for 2 days with their GPS-accelerometer before attempting to recapture them to remove the GPS-accelerometer and re-weigh the birds using the same technique as described above. We calibrated all the GPS-accelerometers in the field prior to deployment following the ‘6–O’ method described in Garde et al. (2022), adjusting the amplitude and offset of each axis (*x*, *y*, *z*) so that all units facing the same direction read the same vectorial sum (see Garde et al., 2022).

For the DLW birds, we had additional manipulations following the protocol for the two-sample method (Schultner et al., 2010). Specifically, upon initial capture where we installed the GPS-accelerometer, we drew a ‘background’ blood sample from the brachial vein (hereafter referred to as background blood sample; 25 G needle) and injected 0.5 ml of DLW into the abdomen (identified easily by the brood patch; 27 G needle). Although we released both control and DLW birds for 2 days with their GPS-accelerometer, DLW birds were recaptured 1.5–3 h after their initial release to obtain a second blood sample (hereafter referred to as initial blood sample) following the same technique as described above. This manipulation was very quick (usually under 3 min) and we released birds immediately after. For birds that we were not able to recapture during the 1.5–3 h time window, we did not obtain an initial blood sample. When completing our final recapture, where we removed the GPS-accelerometers, we drew a third blood sample (hereafter referred to as final blood sample). For all blood samples, we immediately flame-sealed the whole blood in glass capillaries (44.7 μl) and stored the samples at room temperature for later analysis.

All work was approved by the McGill Animal Care Committee (protocol 2016-7814), under state permit 21-089 issued by the Alaska Department of Fish and Game and federal permit MB33779 issued by the US Fish and Wildlife Services.

### Effect of DLW manipulations

We assessed the impact of additional manipulations of the DLW group on the bird's body condition. As we anticipated a negative effect (i.e. loss of mass), we conducted a one-tailed paired *t*-test (as we expected DLW birds to have a greater mass loss than non-DLW birds) and compared body mass at deployment and at retrieval between control and DLW groups among and within the different breeding stages. We conducted additional analyses to distinguish the effects of GPS-accelerometry deployment, if any, on breeding success (see Supplementary Materials and Methods, ‘Tag effects’ for more details).

### Behavioural classification

**Fig. 1.**
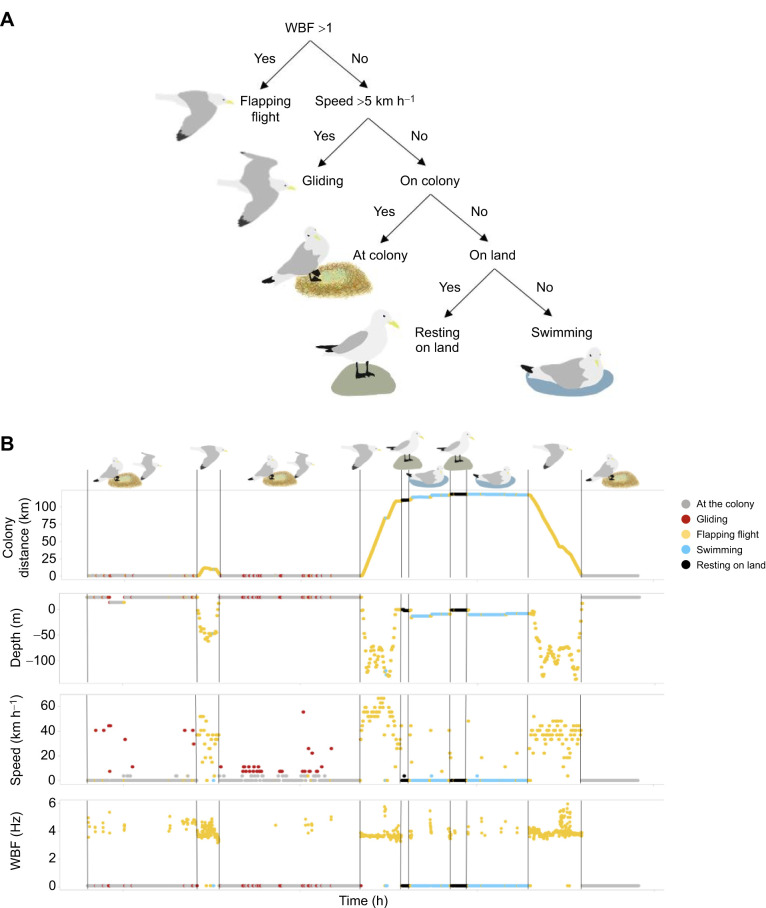
**Behavioural classification using a Hidden Markov Model.** (A) Behavioural classification using a Hidden Markov Model based on wing beat frequency (WBF), ground speed, presence/absence at the colony and presence/absence on land to classify behaviours into five broad categories (flapping flight, gliding, at the colony, resting on land and swimming). (B) An example track with classification shown over the course of a 24 h day.

To determine activity-specific metabolic rate, we created a behavioural time budget (hereafter time–activity budget). Using accelerometry and GPS data, we classified the kittiwakes' behaviour into five broad types: at the colony, resting on land, swimming, flapping flight and gliding. We classified behaviours using a Hidden Markov Model using the momentuHMM package (McClintock and Michelot, 2018) in RStudio (https://posit.co/download/rstudio-desktop/) based on four predictor variables (Fig. 1, Table 1); wingbeat frequency (WBF; Hz), presence at the colony, presence on land and ground speed (km h^−1^). See Table 1 for the complete description and distribution for these variables. We averaged all variables over 10 s intervals as Hidden Markov Models require data at equally sampled intervals. We interpolated GPS location, sampled every 3 min, to match the 10 s intervals.

**Table 1.**
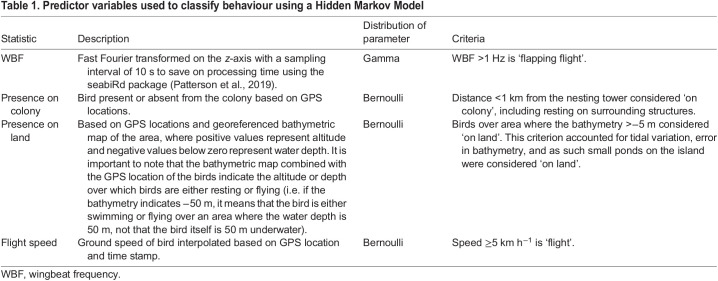
Predictor variables used to classify behaviour using a Hidden Markov Model

Using our classification, we obtained time–activity budgets, as a percentage of time spent in each behaviour per day, for all individuals, which we then converted into time–energy budgets. For each of the five behaviours, we also calculated activity-specific vectorial dynamic body acceleration (VeDBA) using the getDBA function from the seabiRds package (Patterson et al., 2019) with a running mean of 10 s using corrected accelerometry data (see Supplementary Materials and Methods, ‘Supplementary method for vectorial dynamic body acceleration correction’ and Fig. S1 for more details), which we then averaged across each day. It is important to note that although we exclusively targeted males during our study, two incubating individuals were misidentified as males. We decided to include those individuals as it has been shown that incubating males and females have similar activity budgets (Tremblay et al., 2022).

We ran an ANOVA (type II) using the *car* package (Fox and Weisberg, 2019) to test for the direct effects of breeding stage on time–activity budgets. If the ANOVA rendered significant results (α=0.05), we also conducted an analysis of least-squares means (LSM) as a *post hoc* test using the *emmeans* package (https://CRAN.R-project.org/package=emmeans). We reported data as means±s.e.m. All analyses were done in R 4.4 (http://www.R-project.org/).

### Measuring isotope ratios from DLW birds

To measure the ratio of DLW isotopes in the bird's blood, we extracted the water from the blood samples and analysed isotope ratios using a Liquid Water Isotope Analyzer (LWIA; model GLA430 LWIA-91, Los Gatos Research, San Jose, CA, USA; technique demonstrated at: https://www.youtube.com/watch?v=NvIHk_50fYw). We distilled the blood in glass Pasteur pipettes (Fisherbrand™ Disposable Borosilicate Glass Pasteur Pipets K63B1367820D 9 inch; 1 Case 1400 pcs; catalogue no. 13-678-20D) which were flame sealed, and placed them on a hot plate for 48 h. Once distilled, we transferred the water into glass vials for analysis using the LWIA. To prevent memory effects (i.e. where the enrichment level of a given sample affects the measurement of the following sample), we analysed samples with different enrichment levels separately (background, initial and final samples). Additionally, we set the LWIA to conduct five preparatory injections prior to conducting the five measured injections and used the average value of the five measured injections to estimate the ratio of H^2^/H^1^ and O^18^/O^16^. For each run, we included a low and a high standard to correct the measured values, thus minimizing error due to variation between batches.

### Calculating CO_2_ production and DEE

Once we had measured the DLW isotope ratio in the bird's blood, we converted ratios of H^2^/H^1^ and O^18^/O^16^ to estimates of CO_2_ produced over the length of the deployment. We first estimated the mean isotope turnover rate for oxygen and deuterium (*k*_o_ and *k*_d_) in parts per million per hour (ppm h^−1^), using the following formula (Speakman, 1997):
(1)

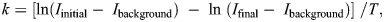
where *I*_background_, *I*_initial_ and *I*_final_ correspond to background, initial and final isotope ratios, respectively, in ppm, and *T* corresponds to the time between the initial and final DLW sample in decimal hours. As we used the two-sample method, we made the assumption that the time at initial sampling corresponds to the time at equilibrium (Speakman, 1997).

Next, we estimated the isotope dilution space of oxygen and deuterium (*N*_o_ and *N*_d_) in the bird's body using the following equation:
(2)

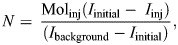
where Mol_inj_ corresponds to the moles of heavy water injected into the bird, *I*_initial_ and *I*_background_ correspond to initial and background isotope ratios in ppm, and *I*_inj_ corresponds to the estimate of injectate enrichment in ppm. To estimate the body water pool, we assumed that the average between the initial measured dilution space of O^18^ (*N*_o_) and the estimated final dilution space corresponds to the pool size (*N*).

We then estimated the rate of CO_2_ production (*r*_CO2_, mmol CO_2_ h^−1^) using Eqn 3 (equation 7.17 of Speakman, 1997):
(3)

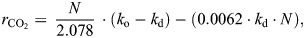
where *N* corresponds to the pool size, and *k*_o_ and *k*_d_ correspond to the turnover rate of the isotopes (equation 7.17 of Speakman, 1997). We converted mmol of CO_2_ into ml of CO_2_ per hour by multiplying by 22,400 (Speakman, 1997). Finally, we converted rates of CO_2_ production into daily energy expenditure of kittiwakes measured using doubly labelled water (DEE_DLW_; kJ day^−1^), using an average caloric equivalent from a multi-year study on kittiwakes (27.63 J ml^−1^ CO_2_; Welcker et al., 2013):
(4)

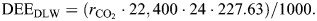
To control for the effect of body mass (larger lean body mass would have higher resting and flight costs; Halsey and Bryce, 2021) and consequently VeDBA (heavier birds would have a lower VeDBA for the same force generated; Gleiss et al., 2011), we first standardized the daily energy expenditure of kittiwakes (in kJ day^−1^) to a body mass of 450 g using the following formula (see Supplementary Materials and Methods, ‘Supplementary method for mass correction’):
(5)

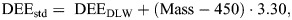
where DEE_DLW_ (in kJ day^−1^) is from Eqn 4 and Mass corresponds to the average of the kittiwake's mass at deployment and retrieval (in g). We then used DEE_std_ in time–energy budget models.

### DLW–behaviour calibration and activity-specific metabolic rate

We first tested whether time–energy budget or DBA approaches best describe the differences in DEE_DLW_ by comparing a full model of time–energy budget (Table 2, model 1) with a full model of activity-specific DBA (Table 2, model 2). We then used a backward stepwise model selection approach to determine which set of activity-specific metabolic rates best describe the variation in DEE_DLW_ and whether some behaviours could be merged to ‘share’ coefficients by including interaction terms (Stothart et al., 2016). To obtain activity-specific metabolic rates and DBA–DEE coefficients, we generated a series of general linear models using the MuMIn package (https://CRAN.R-project.org/package=MuMIn) with DEE_DLW_ as our dependent variable. Using the MuMIn package, we compared models based on their Akaike information criterion corrected for small sample size (AICc; Table 3). We report models with 2 ΔAICc only. This was done using the dredge function by starting with the full model then iteratively combining any two behaviours to determine whether that reduced the AIC; if it did, then we tried a successive iteration with those two behaviours merged and merging all other combinations of two behaviours. For all models that were within 2 ΔAICc (Burnham and Anderson, 2002), we tested for multicollinearity of predictors using variance inflation factors (VIF) and discarded models with VIFs >10 (Vittinghoff et al., 2011). Once we identified the best predictors of energy expenditure from our best model (Table 3, model 2), we extracted estimates for each covariate in the model to obtain activity-specific metabolic rates (in kJ day^−1^) and converted them to kJ day^−1^ g^−1^ by dividing the estimates by 450 g, and similarly calculated coefficients for DBA–DEE relationships. Values are also reported as multiples of BMR (3.49 W; Elliott et al., 2013b). We report only the models with 2 ΔAICc from the best model for the model selection.

**Table 2.**
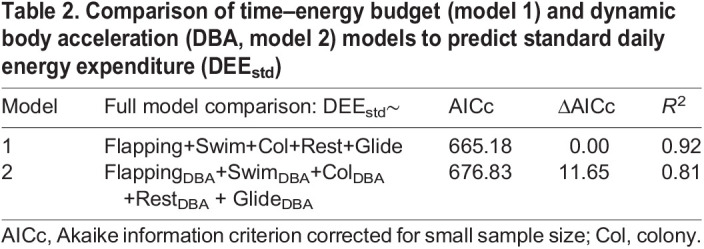
Comparison of time–energy budget (model 1) and dynamic body acceleration (DBA, model 2) models to predict standard daily energy expenditure (DEE_std_)

**Table 3.**
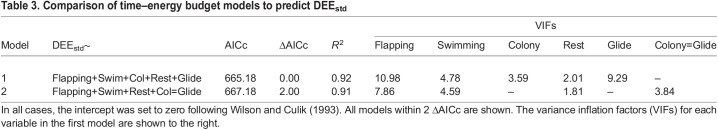
Comparison of time–energy budget models to predict DEE_std_

### Estimates of energy expenditure

Using our activity-specific metabolic rates extracted from the model selection for time–energy budget models, we estimated DEE for all DLW and control birds (*n*=80 total) using their time–energy budgets obtained from the GPS-accelerometry data. We used the following formula to obtain estimates of DEE (Stothart et al., 2016):
(6)

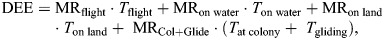
where MR (in kJ g^−1^ day^−1^) corresponds to the activity-specific metabolic rate of each behaviour (time at the colony and gliding pooled together as the best model based on AICc and VIFs showed that they predicted energy expenditure best when pooled as a single predictor), and *T* corresponds to the proportion of time spent in a given behaviour (% per day).

To allow for a comparison between our calibration and that of Jodice et al. (2003), we converted their activity-specific metabolic rates measured in ml CO_2_ g^−1^ h^−1^ to kJ g^−1^ day^−1^ using the same caloric equivalent as described in our methods (see Table 4 for more details). To obtain an estimate for flapping flight using Jodice et al.’s (2003) values, we averaged their activity-specific metabolic rate for both ‘commuting flight’ and ‘searching flight’ (Table 4), and averaged ‘nest attendance’ and ‘loafing near colony’ to obtain an estimate for both resting and gliding (Table 4). Similarly, to compare our values with those of Collins et al. (2016), we converted their values (in kJ h^−1^ for a 365 g individual) to kJ g^−1^ day^−1^ (Table 4). See Table 4 for activity-specific metabolic rates obtained from Jodice et al. (2003) and Collins et al. (2016) for each of our behavioural categories, in kJ g^−1^ day^−1^. For each individual bird, we calculated DEE (from Eqn 6) using ours, Jodice et al.’s (2003) and Collins et al.’s (2016) activity-specific metabolic rates to compare the methods.

**Table 4.**
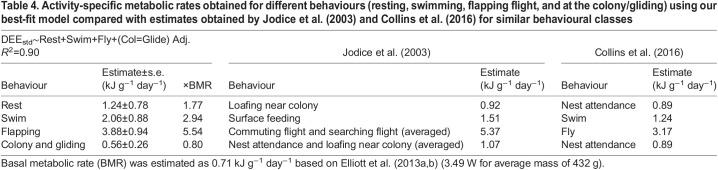
**Activity-specific metabolic rates obtained for different behaviours (resting, swimming, flapping flight, and at the colony/gliding) using our best-fit model compared with estimates obtained by** **Jodice et al. (2003)** **and** **Collins et al. (2016)** **for similar behavioural classes**

We modelled DEE estimated from time–energy budgets obtained from each of the three papers (present study; Jodice et al., 2003; Collins et al., 2016) against DEE measured from DLW. We also compared DEE against breeding stage to see whether breeding stage had a significant impact on DEE. We ran an ANOVA (type II) using the *car* package (Fox and Weisberg, 2019) to test for the direct effects of the breeding stage on DEE measured from DLW and activity budgets. If the ANOVA rendered significant results (α=0.05), we also conducted an analysis of LSM as a *post hoc* test using the *emmeans* package (Russell, 2021). We reported data as means±s.e.m. All analyses were done in R v.4.4. (http://www.R-project.org/).

## RESULTS

Out of 93 kittiwakes we deployed GPS-accelerometers on, we retrieved 92 units and obtained 90 usable tracks spread between pre-laying (*n*=20 DLW, 10 controls), incubation (*n*=34 DLW, 10 controls) and chick rearing (*n*=16 controls). We excluded 10 birds for which we did not obtain a ‘6–O’ calibration in the field, for a total of 80 GPS-accelerometry deployments, out of which 49 birds had been injected with DLW. Out of the 55 birds injected with DLW, we recaptured 54 individuals. We excluded one individual which had final levels of O^18^ within 2% of background levels (Speakman, 1997), one individual whose final sample was destroyed in transport, and the two females from the DLW analyses (though we did include their time–energy budgets), leaving us with a total of 50 individuals from pre-laying (*n*=17) and incubation (*n*=33) (Table S2).

### Effect of DLW treatment

Deployments lasted on average 58.9±2.3 h (range: 39.1–122.2 h). Body mass decreased over the length of the deployment for birds injected with DLW, with an average mass loss of 11.73±4.57 g (paired *t*-test, *t*_48_=2.57, *P*=0.007) between deployment and retrieval. We found no effect of deployment on body mass in control birds (−4.64±5.87 g, paired *t*-test, *t*_27_=0.79, *P*=0.218).

### Time–energy budgets

**Fig. 2.**
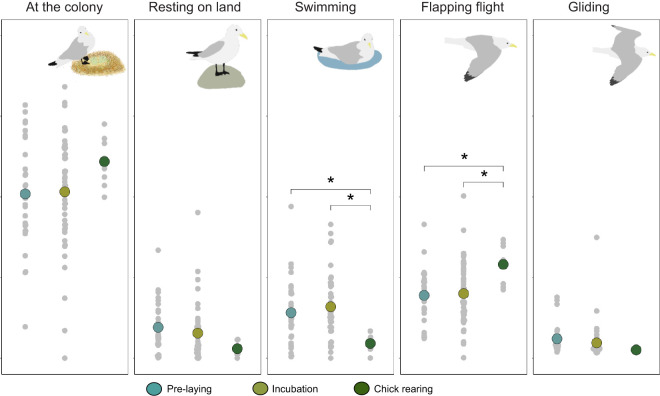
**Activity budgets of 80 kittiwakes throughout the breeding season.** Significantly different groups are identified with an asterisk.

Based on our time–energy budgets, kittiwakes varied their time spent in flapping flight (*F*_2,77_=3.80, *P*=0.03) and swimming (*F*_2,77_=4.68, *P*=0.01) throughout the breeding season. Kittiwakes increased their time spent in flapping flight during chick rearing compared with pre-laying and incubation (Table S1; see Supplementary Materials and Methods, ‘Foraging behaviour’ and Fig. S2). On average, birds spent 32±2% of their time using flapping flight in chick rearing compared with 25±1% in pre-laying and 22±1% in incubation (Fig. 2). In contrast, kittiwakes decreased their time spent swimming during chick rearing (Table S1), with an average of 5±2% compared with 14±2% in pre-laying and 16±1% in incubation (Fig. 2). Time spent at the colony (53±16%) did not vary across the breeding stages. Similarly, time spent resting on land (8±8%) did not vary across the breeding season. Gliding occupied 4±2% of the kittiwakes' time and did not vary across the breeding stages.

### DLW–behaviour calibration and activity-specific metabolic rate

The top model based on time–energy budgets better predicted energy expenditure than the top VeDBA model (Table 2). When we only considered time–energy budget models, and compared models with and without different activities being pooled and breeding stage, both the full model (including time spent in flapping flight, swimming, resting on land, time spent at the colony and time spent gliding as separate terms) and the same model with time at colony and gliding flight pooled were equally parsimonious (ΔAICc=2.0 or lower; Table 3). However, we discarded the full model (Table 3, model 1) because of the VIF being at or nearly 10 for two activities, implying that time spent in flapping flight and in gliding are correlated with one another and thus the coefficients cannot be separately estimated in the full model. Breeding stage was not included as a predictor in any of the most parsimonious models.

Based on the activity-specific metabolic rates derived from time–energy budgets, kittiwakes spent the most energy per day during flapping flight, followed by swimming, resting on land and at the colony and gliding (Table 3).

### Estimates of DEE

**Fig. 3.**
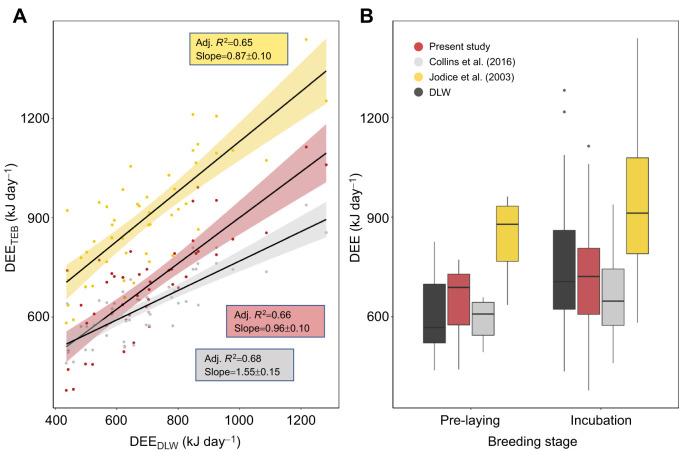
**Comparison of daily energy expenditure (DEE) in 49 kittiwakes.** DEE was measured using time–energy budgets (TEB) using activity-specific metabolic rates from the present study, Collins et al. (2016) and Jodice et al. (2003) and is shown in relation to (A) energy expenditure measured using doubly labelled water (DLW) and (B) per breeding stage, where dark grey boxes represent DEE estimated using DLW. Box plots show median, upper and lower quartiles and 1.5× interquartile range (circles are outliers).

DEE for our birds estimated from our calibration coefficients was similar to DEE for our birds estimated with the model coefficients developed by Jodice et al. (2003) and Collins et al. (2016; Fig. 3; see Table 4 for activity-specific coefficients used for calculations). Estimates obtained using Jodice et al. (2003) activity-specific metabolic rate coefficients consistently yielded higher estimates of DEE, and those estimated using Collins et al., (2016) coefficients yielded lower estimates of DEE for the same individuals.

## DISCUSSION

Using GPS-accelerometry and DLW techniques, we obtained activity-specific metabolic rates to estimate DEE in breeding black-legged kittiwakes. DEE measured via DLW correlated well with time–energy budgets (*R*^2^=0.66, Fig. 3), as well as with a previous time–energy budget calibration for kittiwakes (Fig. 3; Jodice et al., 2003). Moreover, our time–energy budget approach provided similar differences in DEE by stage as previous studies (Figs 2 and 3), supporting our contention that these provide biologically informative models. Thus, we show that, once calibrated, GPS-accelerometers can be used to measure DEE across different activity states.

As per our first hypothesis, time–activity budgets outperformed DBA in predicting energy expenditure. This is intriguing as all previous studies on seabirds examining correlations between DEE, DBA and time budgets found that DBA outperformed time budgets (murres: Elliott et al., 2013a,b; cormorants: Stothart et al., 2016; dovekies: Ste-Marie et al., 2022; penguins: Hicks et al., 2020, Sutton et al., 2021; gannets: Sutton et al., 2023). We argue that this is partly because some individual kittiwakes spend considerable time (up to 30% from some incubating individuals, Fig. 2) in gliding flight. For species (such as gannets) using flap-gliding flight, DBA may be a useful proxy for the relative amount of flapping per flap-glide phase and thus energy expenditure. In contrast, kittiwakes' gliding primarily occurred continuously for many minutes near the colony with essentially zero DBA and low (but not zero) energy expenditure. Indeed, when we did not include gliding as a separate behaviour, our models were nonsensical with flight having negative values for energy expenditure. Alternatively, the lower performance for DBA may be due to environmental DBA, such as wave action or wind turbulence during gliding that may increase DBA without altering energy expenditure (Wilson et al., 2020).

**Fig. 4.**
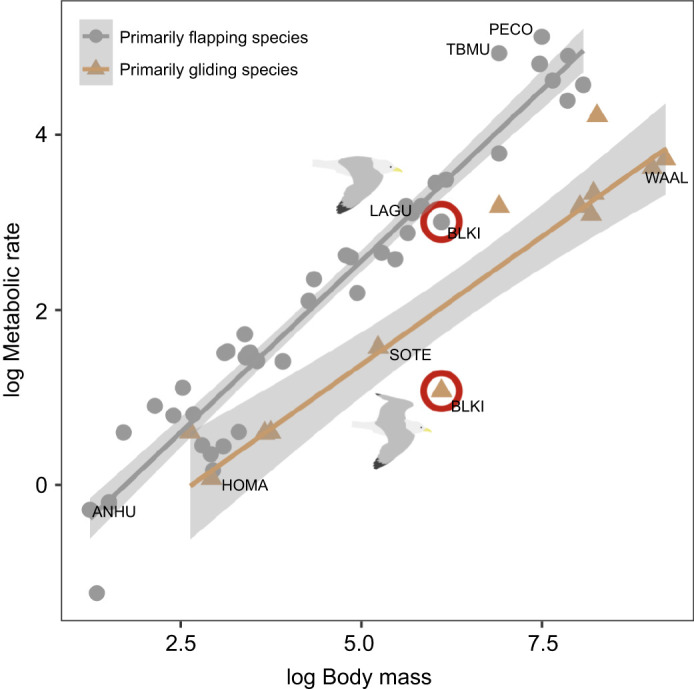
**Variation in flight costs of primarily flapping and gliding bird species.** Flapping and gliding birds were as defined in Guigueno et al. (2019). The metabolic cost of flapping and gliding flight (in watts; kJ h^−1^/3.6) for kittiwakes (450 g average mass) is denoted by the red circles. Body mass and metabolic rate of all species were extracted from Guigueno et al. (2019). Species are denoted by their corresponding 4-letter species code: PECO, *Phalacrocorax pelagicus*; TBMU, *Uria lomvia*; WAAL, *Diomedea exulans*; LAGU, *Leucophaeus atricilla*; BLKI, *Rissa tridactyla*; SOTE, *Onychoprion fuscatus*; HOMA, *Delichon urbica*; ANHU, *Calypte anna.*

Our second hypothesis was also supported: models separating gliding and flapping flight were clearly supported over those that pooled them together (model selection reported in Table 3). To our knowledge, our study is the first to distinguish gliding from flapping flight in kittiwakes using GPS-accelerometry. Although gliding occupies a small portion of the kittiwakes' time–energy budgets, pooling flight and gliding into a single behavioural category could lead to errors such as overestimating energy expenditure, as the cost of gliding/resting at the colony is ∼6.9 times lower than the cost of flapping flight (Table 3). Energy expended when gliding is statistically indistinguishable from the energy expended when at the colony (i.e. nest attendance), rather than the energy spent in flapping flight (Table 3). Although our estimated cost of gliding for kittiwakes appears low compared with the metabolic costs in other species (Fig. 4), it is important to note that the metabolic rates shown in Fig. 4 do not exclude flapping flight from the gliding bouts, but rather show the overall cost of flight in species that use gliding (more than 5% of the time spent in flight; see Guigueno et al., 2019). The cost of flapping flight in kittiwakes is similar to that of other gulls such as laughing gulls (*Leucophaeus atricilla*). Heart rate increases ∼30% (albatrosses; Sakamoto et al., 2013) or ∼100% (gulls; Brown et al., 2022) between gliding and flapping flight, but because the relationship between energy expenditure and heart rate may not be directly proportional between activities (Green, 2011), these changes may be similar to the absolute differences of 690% that we detected using DLW. On top of identifying key metabolic differences between gliding and flapping flight, our behavioural classification also identified key metabolic differences among kittiwakes resting on land, on water and at the colony. These results highlight the importance of selecting species-appropriate behavioural categories when estimating energy expenditure based on time–energy budgets (Halsey and Bryce, 2021), as pooling active (flapping) and inactive (gliding) behaviours together can lead to errors.

Although we used males exclusively in this study, we believe that our calibration is valuable for estimating energy expenditure in both breeding males and females. However, our calibration may not be applicable to females during pre-laying because of the high individual variation in energy demands (based on triiodothyronine; Tremblay et al., 2022), likely as a response to egg formation (Whelan et al., 2021; Creelman and Storey, 1991). Otherwise, there is no difference between male and female energy expenditure during incubation and chick-rearing when accounting for behaviour and mass (Tremblay et al., 2022; Thomson et al., 1998). Before accounting for females in future DLW–behaviour calibrations, we suggest that further research is carried out to elucidate the cost of egg formation in pre-laying females. Furthermore, we acknowledge that our calibration for the cost of gliding and colony attendance yielded estimates below published values of BMR for kittiwakes at this colony (3.49 W or 0.71 kJ g^−1^ day^−1^; Elliott et al., 2013b), which were likely increased by handling stress (e.g. Shoji et al., 2013). Thus, we are confident that our DLW–behaviour calibration can yield accurate estimates of energy expenditure for males and females outside the egg formation period.

Additionally, we detected a DLW effect as body mass declined more in DLW birds than in controls, likely as a response to increased stress from the additional handling. As we used the two-sample method (Speakman, 1997) to obtain more accurate measurements of energy expenditure, the additional capture (2–4 min handling time) might have resulted in an increased time spent in flapping flight due to stress (Schultner et al., 2010). Our measurements of energy expenditure from DLW are within the normal range for kittiwakes; hence, we believe that although tagging had an effect on the birds' mass, it did not affect our energy expenditure results significantly (Gabrielsen et al., 1987; Jodice et al., 2003, 2002; Collins et al., 2016). Furthermore, the negative effect of DLW sampling on body mass highlights the need for our calibration in kittiwakes, as using GPS-accelerometers alone did not affect their body mass.

The energy estimates using our calibration coefficients correlate with estimates for the same birds using the coefficients from Jodice et al. (2003) (Fig. 3), which is the most cited calibration to date. Absolute values of energy expenditure differed with our calibration, yielding consistently lower energy estimates than those in Jodice et al. (2003). This difference may be the result of Jodice et al.’s (2003) smaller sample size and use of a mix of behavioural observations and radio telemetry to track behaviour. It may also be due to individual variation between the Middleton Island and Shoup Bay kittiwake colonies (Jodice et al., 2003). Estimates of energy expenditure obtained using our calibration were similar to those of Collins et al. (2016; Fig. 3), who classified behaviour into three categories (flight, resting at colony, resting on water) using only accelerometer data. However, to accurately determine DEE in our study, it was important to separate flapping from gliding flight (models with flight merged did not converge or gave unreasonable results, such as negative values for the cost of flight), which was not possible using Collins et al.’s (2016) method (with our dataset) because flapping flight was not distinguished from rest using the accelerometer data. As our tags were tail mounted, it was more challenging to determine body posture from the accelerometer signal.

In summary, we have created a novel DLW–behaviour calibration for breeding kittiwakes that offers a less restrictive and less invasive approach to measure energy expenditure than other traditional methods (i.e. heart rate loggers). Our calibration will facilitate the estimation of DEE using only GPS-accelerometers, reducing the impact on animals, researcher effort and expenses. Using broad behavioural categories, time–energy budgets can be easily obtained from free-ranging individuals to estimate energy expenditure. Studying energy expenditure using time–energy budgets can provide insight into marine ecosystem health, on top of supplying valuable information on how seabirds adapt to their changing environment. Although studies based on behavioural observations, species distributions and population demographics can provide some cues on the efficiency of some conservation measures, the physiology of an individual or a population provides a much more sensitive approach to studying animal responses (Wikelski and Cooke, 2006). Specifically, our GPS-accelerometer data would allow us to create an energy landscape and delineate key energy hotspots for species conservation.

## Supplementary Material

10.1242/jexbio.247176_sup1 Supplementary information
