# Supplementary material for: Time–energy budgets outperform dynamic body acceleration in predicting daily energy expenditure in kittiwakes, and estimate a very low cost of gliding flight relative to flapping flight
Source: J Exp Biol. 2024 Nov 7;227(21):jeb247176. doi: 10.1242/jeb.247176 (PMC11574358; doi:10.1242/jeb.247176)
Supplement: Supplementary information [file jexbio-227-247176-s1.pdf]

Supplementary Materials and Methods

Supplementary method for mass correction

Due to variation in mass, we looked at the effect of mass on daily energy expenditure for the pooled data, as well as each breeding stage individually. For the pooled data, we considered both power law (Log (DEE) = 1.91 ± 0.45 * Log (Mass) – 5.14 ± 2.80; t_{47} = 4.209; p = 0.0001; R^2 = 0.27) and linear (DEE = 3.30 ± 0.71 * (Mass) – 780 ± 319; t_{47} = 4.65, $p < 0.0001$; R^2 = 0.32) relationships, where significance tests are given for the slopes. Given the variation in body mass between the two stages, we also considered the stages separately. For incubation, both power law (Log (DEE) = 2.03 ± 0.68 * Log (Mass) – 5.89 ± 4.16; t_{30} = 3.00; p = 0.005; R^2 = 0.23) and linear (DEE = 3.61 ± 1.08 * (Mass) – 917 ± 503; t_{30} = 3.33, p = 0.002; R^2 = 0.27) relationships were significant. For pre-laying, neither power law (Log (DEE) = 0.29 ± 1.16 * Log (Mass) + 4.63 ± 7.00; t_{15} = 0.25; p = 0.81; R^2 = 0.00) nor linear (DEE = 0.57 ± 1.64 * (Mass) + 358 ± 690; t_{15} = 0.35, p = 0.73; R^2 = 0.01) relationships were significant. Given that the power law explained less of the variation than the linear relationship (and the similarity between both relationships), we only included the linear relationship going forward, which we applied on the pooled data given the similarity between the regression lines for incubating and pooled data.

Supplementary method for vectorial dynamic body acceleration correction

VeDBA obtained from calibrated vs. raw accelerometry data correlates tightly, with an R^2 ranging from 0.98 to 1.00 (Fig. S1). Following the calibration, we excluded one outlier from further analysis, likely as a result of a unit malfunction.

Journal of Experimental Biology • Supplementary information

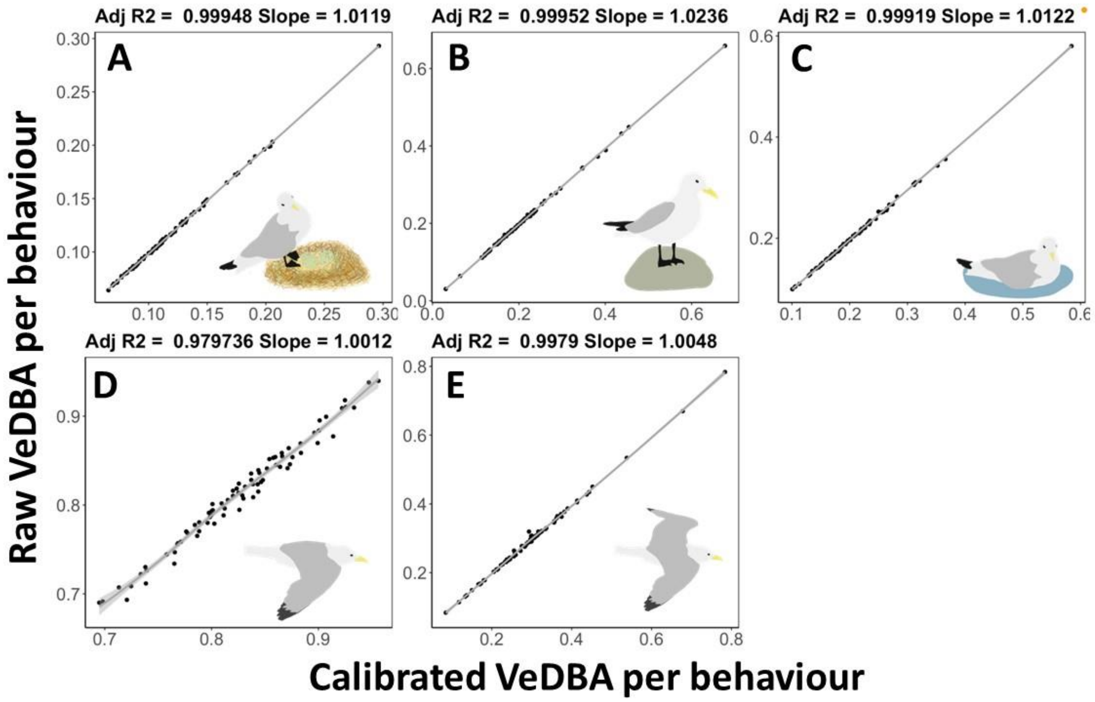

Fig. S1. Comparison of calibrated vs. raw vectorial dynamic body acceleration (VeDBA) of 80 kittiwakes **a)** at the colony, **b)** resting on land, **c)** swimming, **e)** in flapping flight and **f)** gliding.

Journal of Experimental Biology • Supplementary information

Tag effects

Although our experiment did not include non-tagged individuals, we tracked the breeding success of an additional subset of birds that were not tagged with GPS-accelerometer to compare the effect of tagging on breeding success. To assess the impact of tag deployment on the bird's reproductive success, we conducted two Fisher's exact tests comparing the hatching success and chick mortality of tagged vs. non-tagged males using the function *fisher.test* from the *stats* package in RStudio (R Core Team 2020). We reported data as mean ± s.e.

We found no significant effect of tag deployment on hatching success ($p = 0.31$, Fisher's exact test) and chick mortality ($p = 0.47$, Fisher's exact test), with on average 65 ± 3 % of chicks hatching and 53 ± 3% of chick mortality in all birds (non-tagged and GPS-tagged birds, including DLW birds).

Breeding stage

We observed a variation in body mass during incubation (average: 463 ± 29 g; range: 412.5 – 536 g) compared with pre-laying (421 ± 19 g; range: 398-463 g). Based on DLW measurements, kittiwakes increased their daily energy expenditure in incubation compared to pre-laying (129 ± 53.8 kJ d^{-1}, $F_{2, 48} = 376.45$, $p = 0.005$; see Table S2). Similarly, estimates of energy expenditure measured using time-activity budgets showed that kittiwakes exhibit significantly higher energy expenditure during incubation and chick-rearing than pre-laying (Table S1, Fig. 6B).There was no difference in energy expenditure estimated using the DLW and time-activity budget methods for kittiwakes in both pre-laying and incubation ($F_{1,125} = 0.21$, $p = 0.65$; Fig.

Journal of Experimental Biology • Supplementary information

6B). Even though our best model did not include a predictor variable for breeding stage, breeding stage did have a significant effect on daily energy expenditure ($F_{2,125} = 6.4$, $p = 0.002$; Fig. 6B).

Foraging behaviour

To assess foraging behaviour, we analyzed GPS data and identified foraging locations and distances based on the birds' residence in space and time (Torres et al., 2017). Prior to conducting our analysis, we cleaned the data and excluded data points that were further than 130 km from the colony and points where it appeared that birds had travelled more than 3 km in 3 minutes, as these values are likely the result of unit malfunction rather than true values. Foraging locations were identified as areas where birds conducted area restricted search (high space and high time), hence excluding areas where the birds were either commuting (low space and low time) or resting (low space, high time; Torres et al., 2017). We mapped kernel density plots of foraging location (50%, 85%, 95%) with *adehabitatHR* (Calenge, 2006) for each breeding stage to account for differences in foraging distance that could impact the birds' daily energy expenditure and time-activity budgets.

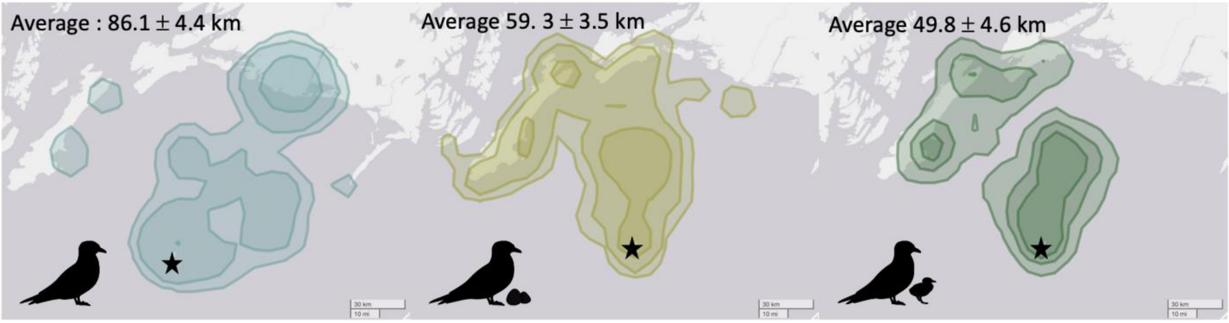

Fig. S2. Foraging location (right, 50%, 85% and 95% kernel density) of 90 kittiwakes across the breeding season.

Kittiwakes foraged at different distances from the colony throughout the breeding season ($p < 0.0001$), with an average foraging distance of 86.1 ± 4.4 km during pre-laying, 59.3 ± 3.5 km in incubation, and 49.8 ± 4.6 km in chick-rearing (Fig. S2). Birds foraged at a greater distance from the colony during pre-laying compared to both incubation and chick-rearing, and foraged the closest to the colony during chick-rearing (Table S1).

Journal of Experimental Biology • Supplementary information

Table S1. Summary of least square means test on linear models looking at the effects of the breeding stage on foraging distance and time-activity budgets and energy expenditure of 90 kittiwakes

Linear Model	*Variables*	*Estimate ± s.e.*	*P value*
Foraging distance ~ Stage (in km)	Pre-laying – Incubation	26.8 ± 5.6	**<0.0001**
	Pre-laying – Chick-rearing	36.3 ± 6.4	**<0.0001**
	Incubation – Chick-rearing	9.5 ± 5.6	**<0.0001**
Time flying ~ Stage (in %)	Pre-laying – Incubation	-1 ± 2	0.97
	Pre-laying – Chick-rearing	-10 ± 4	**0.03**
	Incubation – Chick-rearing	-9 ± 3	**0.03**
Time swimming ~ Stage (in %)	Pre-laying – Incubation	-2 ± 2	0.7
	Pre-laying – Chick-rearing	10 ± 4	**0.04**
	Incubation – Chick-rearing	11 ± 4	**0.009**
DEE ~ Stage (in kJ d^{-1})	Pre-laying – Incubation	-86.8 ± 27.1	**0.005**
	Pre-laying – Chick-rearing	-103.5 ± 43.6	**0.05**
	Incubation – Chick-rearing	-16.6 ± 42.0	0.9

Journal of Experimental Biology • Supplementary information

Table S2. Energy expenditure of kittiwakes measured via doubly-labelled water

Breeding Stage	Sample size (n)	Mean ± s.e. kJ g^{-1} d^{-1}	Mean ± s.e. kJ d^{-1}	Min kJ d^{-1}	Max kJ d^{-1}
Pre-laying	17	1.48 ± 0.08	623 ± 33	439	950
Incubation	33	1.62 ± 0.07	752 ± 36	435	1282
**Total**	50	1.57 ± 0.05	705 ± 27	435	1282

Journal of Experimental Biology • Supplementary information
